# Payments by Patients

**Published:** 1920-10-30

**Authors:** 

**Affiliations:** Chairman, London Committee of the British Hospitals Association. 3 Grosvenor Place, London, S.W. 1


### THE EDITOR'S LETTER-BOX.

**Correspondence on all subjects is invited, but we cannot in any way be responsible for the opinions expressed by our correspondents, who must give their name and address as a guarantee of good faith, but not necessarily for publication. Correspondents are reminded that brevity of style and conciseness of statement greatly facilitate early insertion.**

### Payments by Patients.

*To the Editor of* The Hospital.

Sir,—There has recently been a discussion in the Press on the subject of payment by patients in the voluntary hospitals. The London Committee of the British Hospitals Association considered the question and determined to circulate the following resolution :

"The London Committee of the British Hospitals Association is of opinion that, having regard to the financial position of the hospitals, the time has arrived when the principle of requiring patients to contribute towards the cost of their maintenance should be recommended with a view to its general adoption in London."

The replies received show that practically all London hospitals are now putting into practice the principle supported by my Committee, and that, in many cases, a considerable revenue is being derived from this source. The institution of a flat rate is not favoured as a rule, and experience will no doubt show whether the best results are obtained by adopting that method or by asking each patient to give something, however small, towards the cost of maintenance.

It is to be hoped that this information will encourage the supporters of voluntary hospitals to maintain their subscriptions, since they will now realise that those who receive the benefits of treatment are recognising that they have some responsibility in helping to preserve the voluntary system.—Yours faithfully,

Hambleden,
Chairman, London Committee of
the British Hospitals Association.

3 Grosvenor Place, London, S.W. 1.
October 21, 1920.

### Health and Artistry.

*To the Editor of* The Hospital.

